# *Taxus* tree-ring chronologies from southern England reveal western European hydroclimate changes over the past three centuries

**DOI:** 10.1007/s00382-025-07601-2

**Published:** 2025-01-30

**Authors:** Tatiana Bebchuk, Andy K. Moir, Tito Arosio, Alexander V. Kirdyanov, Max C. A. Torbenson, Paul J. Krusic, Toby R. Hindson, Heidi Howard, Agata Buchwal, Charles A. P. Norman, Ulf Büntgen

**Affiliations:** 1 Department of Geography, University of Cambridge, Cambridge, CB2 3EN UK; 2 Institute for the Environment, Brunel University, London, WC1H 0DG UK; 3 Tree-Ring Services, Oakraven Field Centre, Mitcheldean, Gloucestershire GL17 0EE UK; 4 Siberian Federal University, Krasnoyarsk, 660041 Russian Federation; 5 Department of Geography, Johannes Gutenberg University, 55099 Mainz, Germany; 6 Department of History, Stockholm University, Stockholm, Sweden; 7 Institute of Geoecology and Geoinformation, Adam Mickiewicz University, B. Krygowskiego 10, 61-680 Poznan, Poland; 8 Czech Globe Global Change Research Institute, Czech Academy of Sciences, 60300 Brno, Czech Republic; 9 Department of Geography, Faculty of Science, Masaryk University, 61137 Brno, Czech Republic; 10 Laboratory of Tree-Ring Research, University of Arizona, 1215 E. Lowell Street, AZ 85721 Tucson, USA

**Keywords:** Anthropogenic warming, Climate reconstruction, Dendrochronology, Drought, Hydroclimate, Paleoclimate, Precipitation

## Abstract

Heatwaves and summer droughts across Europe are likely to intensify under anthropogenic global warming thereby affecting ecological and societal systems. To place modern trends and extremes in the context of past natural variability, annually resolved and absolutely dated climate reconstructions are needed. Here, we present a network of 153 yew (*Taxus baccata* L.) tree-ring width (TRW) series from 22 sites in southern England that cover the past 310 years. Significant positive correlations were found between TRW chronologies and both April–July precipitation totals (r > 0.7) and July drought indices (r > 0.59) back to 1901 CE (*p* < 0.05). We used a suite of residual and standard TRW chronologies to reconstruct interannual to multi-decadal spring–summer precipitation and mid-summer drought variability over western Europe, respectively. Our yew hydroclimate reconstructions capture the majority of reported summer droughts and pluvials back to 1710 CE. Clusters of severe drought spells occurred in the second half of the 18th and mid-twentieth century. Our study suggests that the frequency and intensity of recent hydroclimate extremes over western Europe are likely still within the range of past natural variability.

**Supplementary Information:**

The online version contains supplementary material available at 10.1007/s00382-025-07601-2.

## Introduction

A sequence of heatwaves and summer droughts caused unusually high death tolls since 2018 (Vautard et al. 2020), together with exceptional economic losses (García-León et al. 2021), and extensive forest dieback over much of Europe and the Mediterranean region (Senf and Seidl 2020). Recent central European summer droughts have been decsribed as unprecedented in the context of the past 2000 years (Büntgen et al. 2021), and multi-year drought episodes are projected to occur more frequently in the future (Van Der Wiel et al. 2023). Prolonged dry periods can trigger tree mortality from local to continental scales (Senf et al. 2020), shape the structure and composition of forest ecosystems (Schuldt et al. 2020), and even drive species extinction (Feyen et al. 2020). Meanwhile, extreme precipitation events have become more frequent and are likely to intensify in western Europe under global warming (Kendon et al. 2023; Watts et al. 2015). Pluvials may impact freshwater quality (Whitehead et al. 2009), exacerbate water-spread diseases (Allan 2011), and cause flash floods (Dale 2021), with western Germany in July 2021 and eastern Spain in October 2024 being two recent catastrophic examples. To better understand the frequency and intensity of modern (hydro)climatic extremes and constrain climate model simulations, we need to contextualize recent conditions against the full range of past variations (Esper et al. 2024; Tejedor et al. 2024).

Systematic meteorological measurements in western Europe began as early as the seventeenth century (Jones and Bradley 1992). Among the longest instrumental records worldwide are the Central England Temperature (CET) series beginning in 1659 (Manley 1974), and the English precipitation series recorded by volunteers in the form of the “Ten Years Rainfall sheet” since 1677 (Hawkins et al. 2023). However, the reliability of these records is low due to to the scarcity of data points and the imperfection of early meteorological observations (Wigley et al. 1984b), not rectified until the 1873 Vienna Meteorological Congress when widespread and systematic development of a European meteorological network began (Jones and Bradley 1992). Therefore, annually resolved and absolutely dated proxy-based climate reconstructions are required to reliably extend these early instrumental records back into the pre-industrial period and help fill spatial gaps in the observational network (Frank et al. 2007).

Tree-ring chronologies can provide high-resolution climate reconstructions for the past millennium (Esper et al. 2016; Ljungqvist et al. 2020). To date, more than 150 tree-ring chronologies of different lengths have been developed and published for western Europe (https://www.ncei.noaa.gov). Oak TRW chronologies from central Europe (Büntgen et al. 2011), southern Germany (Land et al. 2019), and southern England (Cooper et al. 2013; Wilson et al. 2013) were used for millennium-long spring–summer precipitation reconstructions. Further to these regional studies, the Old World Drought Atlas (OWDA; Cook et al. 2015) utilises 106 local to regional tree-ring chronologies, of which 19 are located in western Europe. The OWDA provides an exceptionally valuable, near-millennium-long, high-resolution gridded summer drought reconstruction. Many of the individual chronologies used in the OWDA, however, end in the late twentieth century. Moreover, due to mild and humid oceanic climate conditions in western Europe, tree growth is typically not limited by a single dominant climate factor. Owing to this, the growth coherency and the proxy-target agreement of TRW chronologies from western Europe are relatively weak (Ljungqvist et al. 2020). Tree-ring stable isotopes (TRSI) were shown to contain strong hydroclimate signals even under less constrained growth conditions (McCarroll and Loader 2004), enabling spring–summer precipitation and summer drought reconstructions to be developed for the UK (Loader et al. 2020; Rinne et al. 2013; Young et al. 2015), France (Labuhn et al. 2016), and central Europe (Büntgen et al. 2021; Freund et al. 2023). Despite the great dendroclimatological achievements of the past decades, the quest for additional tree species that contain distinct climate signals, and possibly allow composite chronologies to be developed over long periods of time, continues.

European yew (*Taxus baccata* L.) is a long-lived conifer species characterised by irregular growth increments (Thomas and Polwart 2003). To date, there are only 13 dendroclimatological studies of *T. baccata* worldwide (Table 1), out of which four are from Asia and nine from Europe. The existing yew TRW chronologies typically integrate up to 30 samples, span between 100 and 400 years, and have recent end dates between 1990 and 2015 CE. Growth-climate analyses revealed either spring–summer precipitation totals or winter–spring temperatures to be the dominant climate factors controlling yew ring width formation. Yet, none of these pioneering studies have resulted in a climate reconstruction. Meanwhile, there is recent evidence from eastern England that the species can possibly allow chronologies to be developed back into the mid-Holocene (Bebchuk et al. 2024). Hundreds of well-preserved subfossil yew trunks were found in peat-rich soils in the east of England, and combined dendrochronological and radiocarbon dating resulted in an 800-year long TRW chronology spanning the fifth millennium BP. To facilitate future dendroclimatic explorations of this subfossil yew dataset, a better understanding of climate factors influencing yew growth in the same or nearby region is needed.

**Table 1:** Overview of *Taxus baccata* tree-ring chronologies and their climate signals worldwide

Publication	Region	Length, years	Span, years CE	N of samples	Temperature signal	Precipitation signal
Parsapajouh et al. (1986)	Iran	455	1545−2000	11	Feb−March	May−June
Moir (1999)	UK	303	1690−1992	14	Jan−Feb, negative July	Feb−July
	Bulgaria	96	1902−1998	11		June
Yadav and Singh (2002)	India, Himalayas	345	1656−2000	18	Negative March−June	
Cedro and Iszkuło (2011)^*^	Poland	218	1790−2007	14	Jan−March, negative July for females, Aug for males	June−July for females
Iszkulo et al. (2011)	Ukraine	85	1922−2007	30	Negative *July, Sep*, Feb−March, *Dec* for females, negative May for males, July for males	April for females
Galvin et al. (2014)	Ireland	204	1805−2007	31	*Nov*−Apr	May−June
Cedro and Cedro (2015)	Poland	219	1790−2008	51	*Dec*−Apr, July−Aug	April
Sedmáková et al. (2017)	Slovakia	117	1898−2015	38	*May−June*, *Nov*−March, negative May−June	June−Sep
Asadulayev et al. (2018)	Russia, Caucasus	159	1851−2010	24	March, Aug−Sep	
Seyfullayev et al. (2021)	Azerbaijan	149	1867−2013	23	*Nov*−March	April
Cedro (2023)^**^	Poland	267	1741−2007	20	*Dec*−March	June
Tayyab et al. (2023)	Pakistan	156	1863−2019	13	Negative April	*February*

Climate signal has a positive correlation unless specified; the months *in italic* are of the previous year. ^*^The statistics for Cedro and Iszkuło (2011) is given for the longest chronology out of 10 presented in the paper. ^**^The statistics for Cedro (2023) is given for the longest chronology out of 34 presented in the paper

Here, we present a network of yew TRW chronologies from southern England, identify their climate signals, and use a network-wide master chronology to reconstruct western European hydroclimate over the past three centuries. We then discuss the frequency and intensity of recent western European droughts and pluvials in the context of reconstructed hydroclimate variability.

## Data and methods

**Fig. 1:**
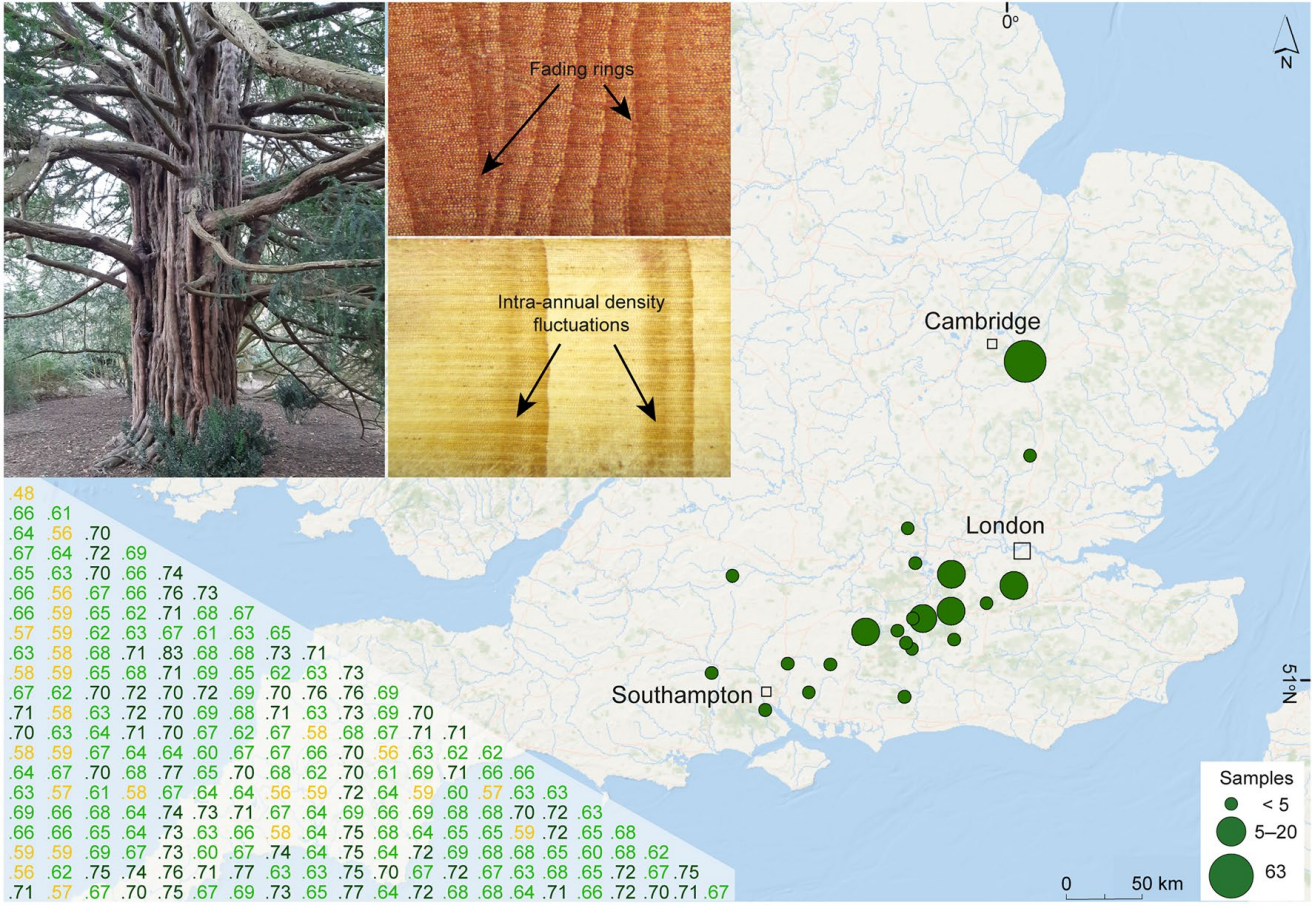
Yew sampling network. The spatial distribution of 22 sites (green cicrcles) with a total of 186 yew trees in southern England. The upper-left inset shows one of the sampled yew trees and anatomical features of yew ring growth: fading (i.e.,wedging) rings and intra-annual density fluctuations (IADF). The correlation matrix in the lower left corner displays the degree of inter-chronology synchronous growth changes, after Visser (2021)

Over several sampling campaigns between 1999 and 2022 (for details see Hindson and Moir 2020, 2021a, b, c, 2022; Moir 1999, 2003a, b, 2004, 2005, 2007, 2010), we collected 5 mm increment core and up to 5 cm thick disc samples from 186 living and dead yew trees at 22 sites in southern England (Fig. 1, Table 2). All samples were polished with up to 800-grit sandpapers, and TRWs were measured at 0.001 mm precision using either the Dendro for Windows programme suite (Tyers 1999), or a Velmex™ measuring system and MeasureJ2X software (Voorhis and Krusic 2006). Since wood anatomical features, including fading (i.e., wedging) rings and intra-annual density fluctuations complicated cross-dating, we preformed additional visual and statistical cross-dating with TSAPWin (Rinn 1996), CDendro (Maxwell and Larsson 2021), and COFECHA (Holmes 1983). A TRW chronology was built for each site that included more than one sample using the ARSTAN software (Cook et al. 2017), and samples with a correlation < 0.5 against the site-specific master chronology were excluded. We then computed a correlation matrix to assess the synchronicity of the site-specific TRW chronologies (or time-series if a site included just one sample), and since no pair of the chronologies correlated < 0.45, all were used to construct a network-wide TRW master chronology (Visser 2021). The master chronology was then detrended with different spline functions ranging from 30 to 250 years of length at 50% frequency cut-off. Two ensembles of the network-wide TRW master chronologies, residual (RES) and standard (STD), were used for subsequent growth-climate relationship analyses.

**Table 2:** Yew sampling sites from northeast to southwest

Location	Site type	Latitude	Longitude	Trees sampled	Trees cross-dated	Length	Timespan
Cambridge	Garden&Park	52.203294	0.111418	70	63	157	1866–2022
Thorley	Churchyard	51.849188	0.14147575	2	2	125	1878–2002
Hedgerley	Churchyard	51.576491	− 0.60090051	1	1	99	1911–2009
Hythe End	Garden	51.444402	− 0.55673532	1	1	230	1760–1989
Hampton Court	Garden	51.403106	− 0.33792584	14	14	283	1710–1992
Savernake Forest	Woodland	51.397964	− 1.6679093	3	2	185	1830–2014
Coulsdon	Woodland	51.359953	0.04244598	13	11	185	1816–2000
Keston	Garden	51.292571	− 0.12262133	1	1	149	1831–1979
Mickleham	Woodland	51.263663	− 0.34009393	8	5	272	1736–2008
Newlands Corner	Woodland	51.235179	− 0.50885861	29	20	247	1775–2022
Guildford	Garden	51.23443	− 0.57133577	1	1	164	1840–2003
Peper Harow	Churchyard	51.187953	− 0.66392499	2	1	133	1874–2006
Farnham	Woodland	51.183356	− 0.85821672	28	20	302	1710–2012
Capel	Churchyard	51.153759	− 0.32030361	2	2	153	1850–2002
Hambledon	Churchyard	51.141961	− 0.61407589	2	1	144	1861–2004
Dunsfold	Churchyard	51.117752	− 0.57508	2	2	155	1849–2003
Winchester	Garden	51.061622	− 1.3316829	2	1	157	1863–2019
West Tisted	Churchyard	51.058396	− 1.0736178	1	1	200	1810–2009
Odstock	Woodland	51.025988	− 1.7946233	1	1	260	1725–1984
Ide	Garden	50.952784	− 1.2022111	1	1	71	1934–2004
Barlavington	Woodland	50.935317	− 0.6223617	1	1	100	1893–1992
Marchwood	Woodland	50.885098	− 1.469574	1	1	309	1710–2018

We analysed growth-climate relationships by correlating the TRW RES and STD chronologies against gridded 0.5 × 0.5° instrumental temperature, precipitation, and the self-calibrating Palmer Drought Severity Index (scPDSI, van der Schrier et al. 2013) obtained from the Climate Research Unit TS 4.07 (CRU, Harris et al. 2020) over the 51−52°N and 2°W−1°E study region in southern England. Pearson’s correlation coefficients were computed for all months and seasonal combinations of the current year of tree growth for the full period (1901−2020), as well as for two independent early/late split periods (1901−1960 and 1961−2020). To further evaluate the temporal stability of the proxy-target agreement, 30-year moving correlation coefficients were computed. We also calculated lagged autocorrelation coefficients to explore possible memory effects in the TRW and climate data. Spatial correlation maps between the chronologies and the climate parameters were computed using the Royal Netherlands Meteorological Institute (KNMI) Climate Explorer (Trouet and Oldenborgh 2013).

We found the TRW residual chronology correlates significantly (*p* < 0.05) with April−July precipitation totals, whereas the standard chronology has high correlations with July scPDSI. We then used a scaling approach to develop two pseudo-independent hydroclimate reconstructions (*R*). The proxy data (*P*) was scaled against the target data (*T*) with the mean (*P*_*mean*_*, T*_*mean*_) and the standard deviation (*P*_*sd*_*, T*_*sd*_) calculated for the full period 1901−2020: ${R}_{i}=\left({P}_{i}-{P}_{mean}\right)/{P}_{sd}*{T}_{sd}+{T}_{mean}$. To estimate the reconstructions’ uncertainty, we calculated three types of error: sample replication error, chronology error, and calibration error. The sample replication error (*E*_*sr*_) was defined as the number of samples per year raised to the power of -1. The chronology error (*E*_*ch*_) was defined as the 97.5% confidence intervals calculated using the bootstrap method, which iteratively produces chronologies with N − 1 series. The *E*_*sr*_ and *E*_*ch*_ are expressed in the units of the chronology and therefore need to be rescaled to the units of the target: ${E}_{i scaled}=\left({P}_{i}-{E}_{sr}-{E}_{ch}-{P}_{mean}\right)/{P}_{sd}*{T}_{sd}+{T}_{mean}$. The calibration error (*E*_*c*_) was computed as the Root Mean Square Error: ${E}_{c}=\sqrt{\sum {\left({T}_{i}-{R}_{i}\right)}^{2}}$. Finally, the minimum and maximum uncertainty intervals of the reconstructions are given by: ${U}_{min}={R}_{i}-{E}_{i scaled}-{E}_{c}/2$ and ${U}_{max}={R}_{i}+{E}_{i scaled}+{E}_{c}$, respectively. The three types of errors were further divided by 2, to keep the uncertainty intervals within the range of possible target variability.

## Results

**Fig. 2:**
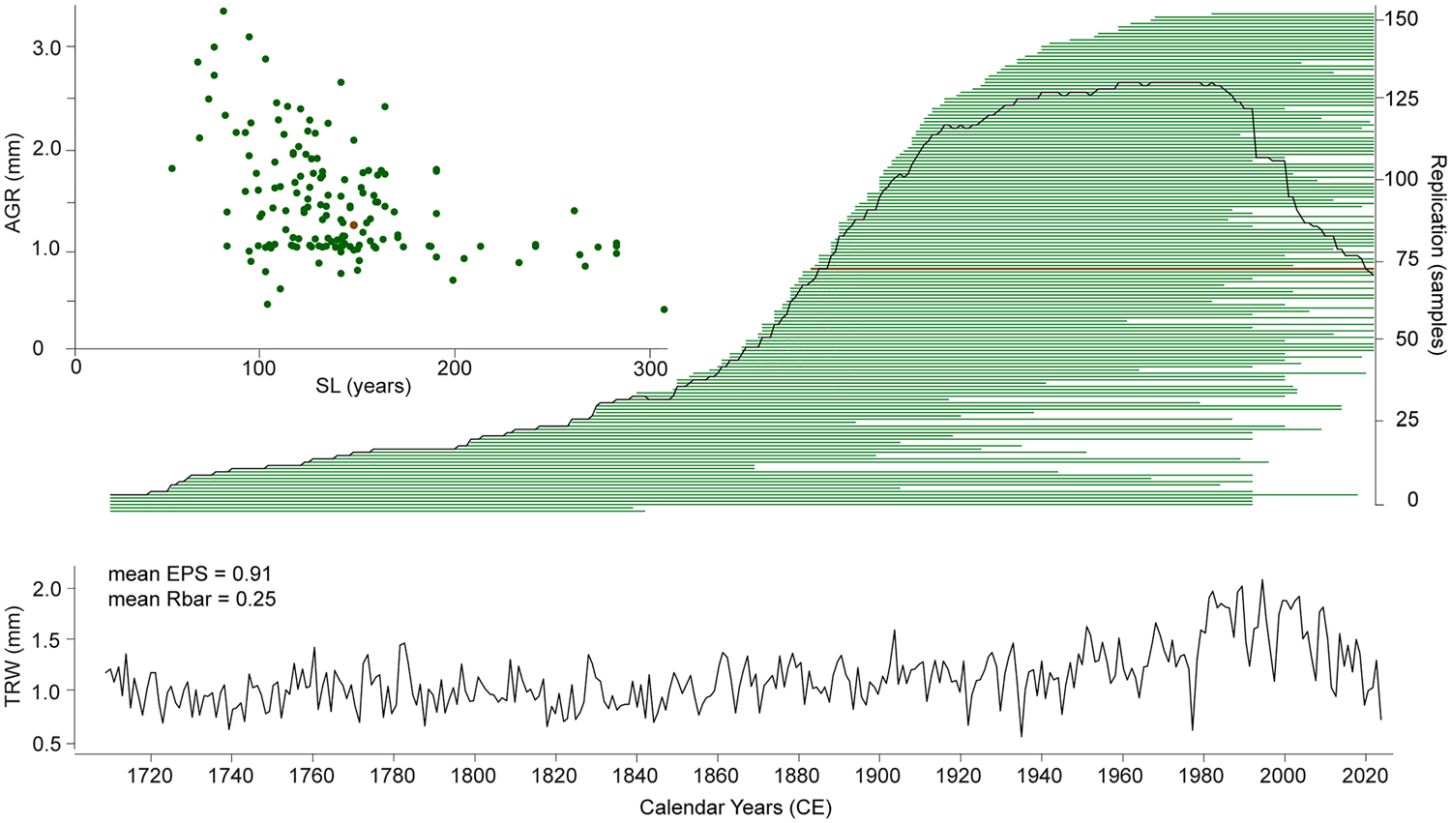
Summary plots of the yew dataset. Relation between segment length (SL, years) and average growth rate (AGR, mm) in the upper-left panel; temporal distribution of 153 yew samples sorted by end dates in green and annual sample replication in black in the upper-right panel. Note that highlighted in brown (a dot and a line) is a tree shown in the photo of Fig. 1. The bottom panel shows the raw tree-ring width yew chronology and its mean expressed population signal (EPS) of 0.91 and a mean Rbar of 0.25, calculated over 50-year segments lagged by 25 years

The yew TRW network from southern England exhibits a high inter-series growth coherency, with cross-chronology correlations ranging between 0.48 and 0.83 (Fig. 1). Only 33 samples out of 186 were not included in the final chronology due to the low correlations. The final composite chronology consists of 153 samples and spans from 1710 to 2020 CE (Fig. 2). The average growth rate of single series (AGR) ranges between 0.3 and 3.4 mm, with a mean of 1.4 mm, and a mean segment length (SL) is 132 years, with the youngest and oldest samples containing 41 and 309 rings, respectively. The chronology has its lowest sample replication of six between 1710 and 1725, followed by a gradual increase to 25 samples over the next 150 years, a peak of 130 samples in the 1970s, and a drop to 70 samples at the most recent end of the chronology. The raw, i.e. non-detrended, chronology exhibits a mean inter-series correlation (Rbar) of 0.25 and a mean expressed population signal (EPS) of 0.91, sufficiently above the accepted threshold of 0.85 (Wigley et al. 1984a).

Correlating the TRW chronologies against gridded instrumental climate data reveals that high spring and summer precipitation totals positively influence yew growth, whereas dry and hot summers result in narrower rings. The RES chronology shows significant (*p* < 0.05) correlations with precipitation totals in April, May, June, and July (AMJJ), yielding the strongest correlation with a sum of all four months. The STD chronology exhibits weaker correlations with precipitation totals compared to the RES chronology. Instead, it shows high correlations with the scPDSI data, which incorporate not only precipitation but also temperature and soil conditions, resulting in a longer memory effect (Wells et al. 2004). Significant correlations between the STD chronology and scPDSI are found from April to December, including all seasonal combinations of these months, with the strongest correlation observed in July.

**Fig. 3:**
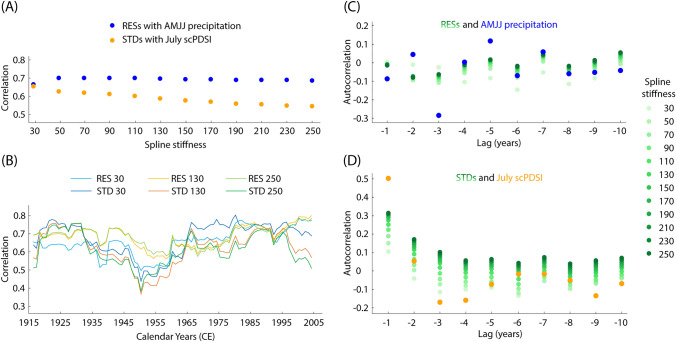
Chronology behaviour under different detrending methods. **A** Correlation coefficients of residual (RES, in blue)/standard (STD, in orange) yew chronologies detrended with a spline stiffness of 30 to 250 years lagged 20 years against gridded (51−52°N, 2°W−1°E) instrumental CRU TS 4.07 0.5 × 0.5° AMJJ precipitation/July scPDSI series. **B** 31-year running correlations of residual/standard chronologies detrended with spline stiffness of 30, 130, or 250 years against AMJJ precipitation/July scPDSI series. **C** Autocorrelations of residual chronologies detrended with spline stiffness of 30 to 250 years lagged 20 years against AMJJ precipitation series. **D** As **C**, but standard chronologies against the July scPDSI series

**Fig. 4:**
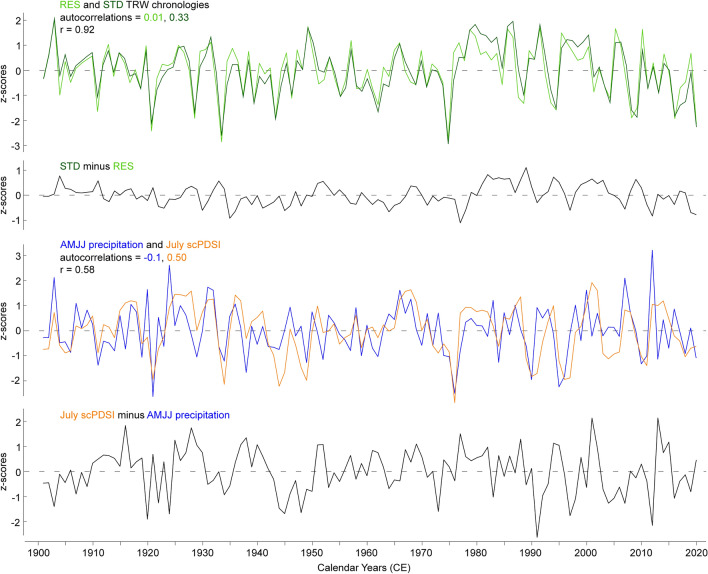
Z-scores of proxy and target data. Yew TRW residual (RES, light green) and standard (STD, dark green) chronologies with their residuals, and gridded (51−52°N, 2°W−1°E) instrumental AMJJ precipitation series (blue) and July scPDSI series (orange) with their residuals for the period 1901−2020

Detrending methods have little effect on the correlations between RES chronologies and AMJJ precipitation totals (Fig. 3A). The correlation increases from 0.67 to 0.71 as the spline stiffness increases from 30 to 70 years, but with a further stiffness increase, the correlation gradually declines to 0.69. In contrast, the STD chronology has its highest correlation of 0.66 with July scPDSI when detrended with a spline stiffness of 30 years. Increasing the spline stiffness leads to a constant decline in correlation down to 0.55. The 30-year running correlation, computed for the chronologies detrended with a spline stiffness of 30, 130, and 250 years (Fig. 3B), exhibits a similar pattern: stable correlations in the range of 0.5−0.8 from 1915 to 1945, followed by a drop to 0.35−0.6 around 1945 and a subsequent rise to original values plateauing around 1965. None of the detrended chronologies have lagged autocorrelations identical to those of the targets (Fig. 3C, D). The autocorrelations of AMJJ precipitation range between −0.1 and 0.1, with a drop to -0.3 at a three-year lag. Although the autocorrelations of the residual chronologies also fall between −0.1 and 0.1, their range in values is narrower (Fig. 3C). The autocorrelations of July scPDSI decrease from 0.5 at the year t-1 to −0.2 at the year t-3, then rises to 0 at t-6, and declines again to −0.15. The STD chronologies follow the same pattern, but each version of STD chronologies has a smaller amplitude, ranging within three decimal points (Fig. 3D). Overall, the analysis of various detrending methods revealed that the pair of RES and STD chronologies detrended with a 30-year spline has the highest mean and the most stable correlations over time. However, such flexible detrending removes possible long-term trends. To preserve long-term variability, the RES and STD chronologies detrended with a spline stiffness of 130 years, which is the mean segment length of the dataset, were used for further climate reconstructions. These chronologies exhibit a mean EPS of 0.95 and 0.93 and a mean Rbar of 0.35 and 0.26, respectively, calculated over 50-year segments lagged by 25 years. The behaviour of pseudo-independent normalised proxies and targets is shown in Fig. 4. The chronologies correlate with each other at 0.9, while the AMJJ precipitation and July scPDSI series correlate at 0.6. The first-order autocorrelation of the RES chronology is lower than that of the STD chronology, following the pattern of the targets. The RES chronology has a higher variance than that of the STD chronology, as well as AMJJ precipitation ranges with a higher amplitude than July scPDSI.

**Fig. 5:**
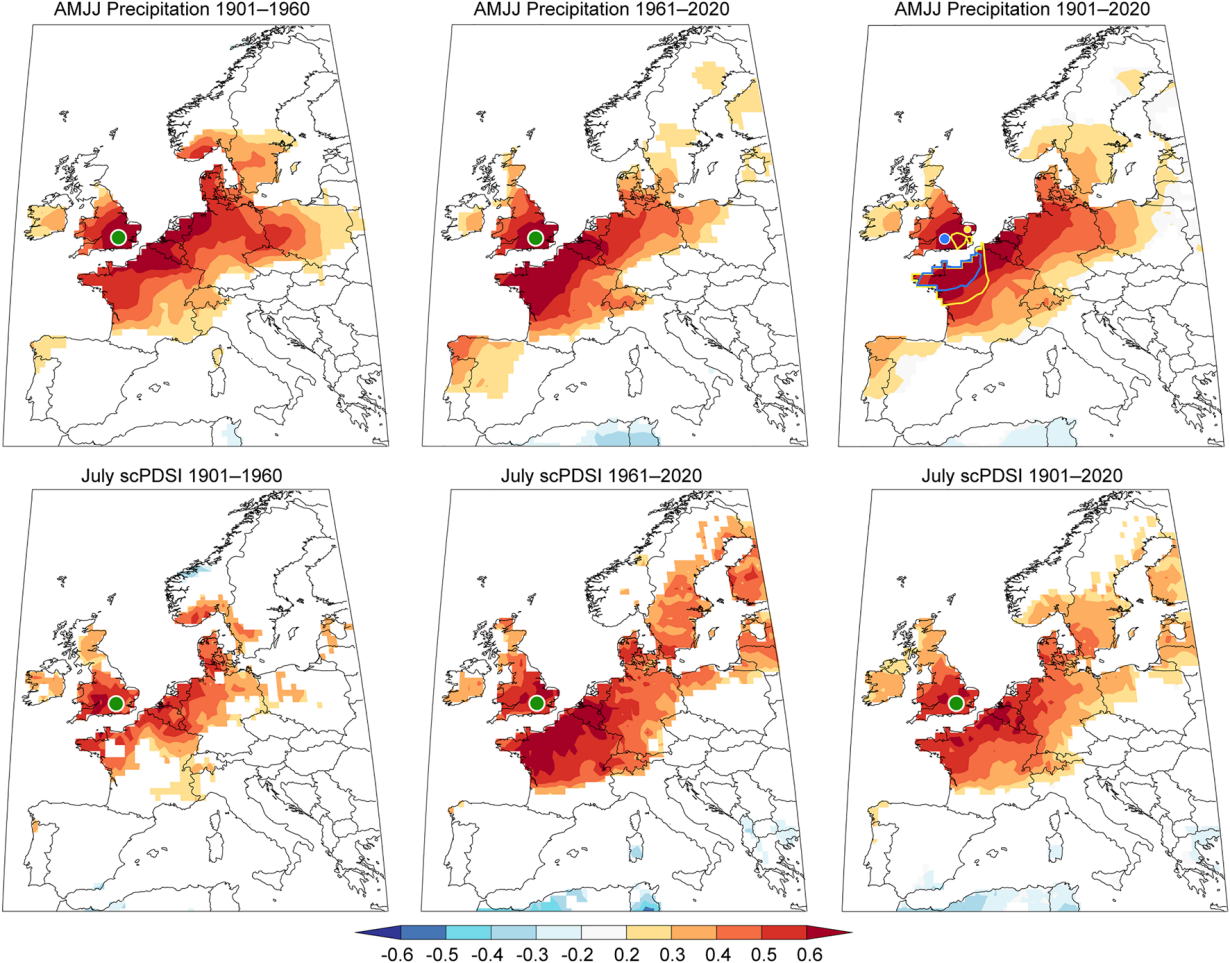
Spatial correlations over Europe. An upper (lower) panel shows spatial correlations of the residual (standard) yew chronology against gridded instrumental AMJJ precipitation (July scPDSI) data. Correlations are computed for two early/late split periods 1901−1960 and 1961−2020 and the full period 1901−2020. All correlations are significant at *p* < 0.1. The green dot denotes the origin of the yew chronologies. In the upper right map, the extent of spatial correlations of 0.5 are shown for Wilson et al. (2012) (blue) and Cooper et al. (2012) (orange) precipitation reconstructions, and the dots represent the proximate source regions of their material

**Fig. 6:**
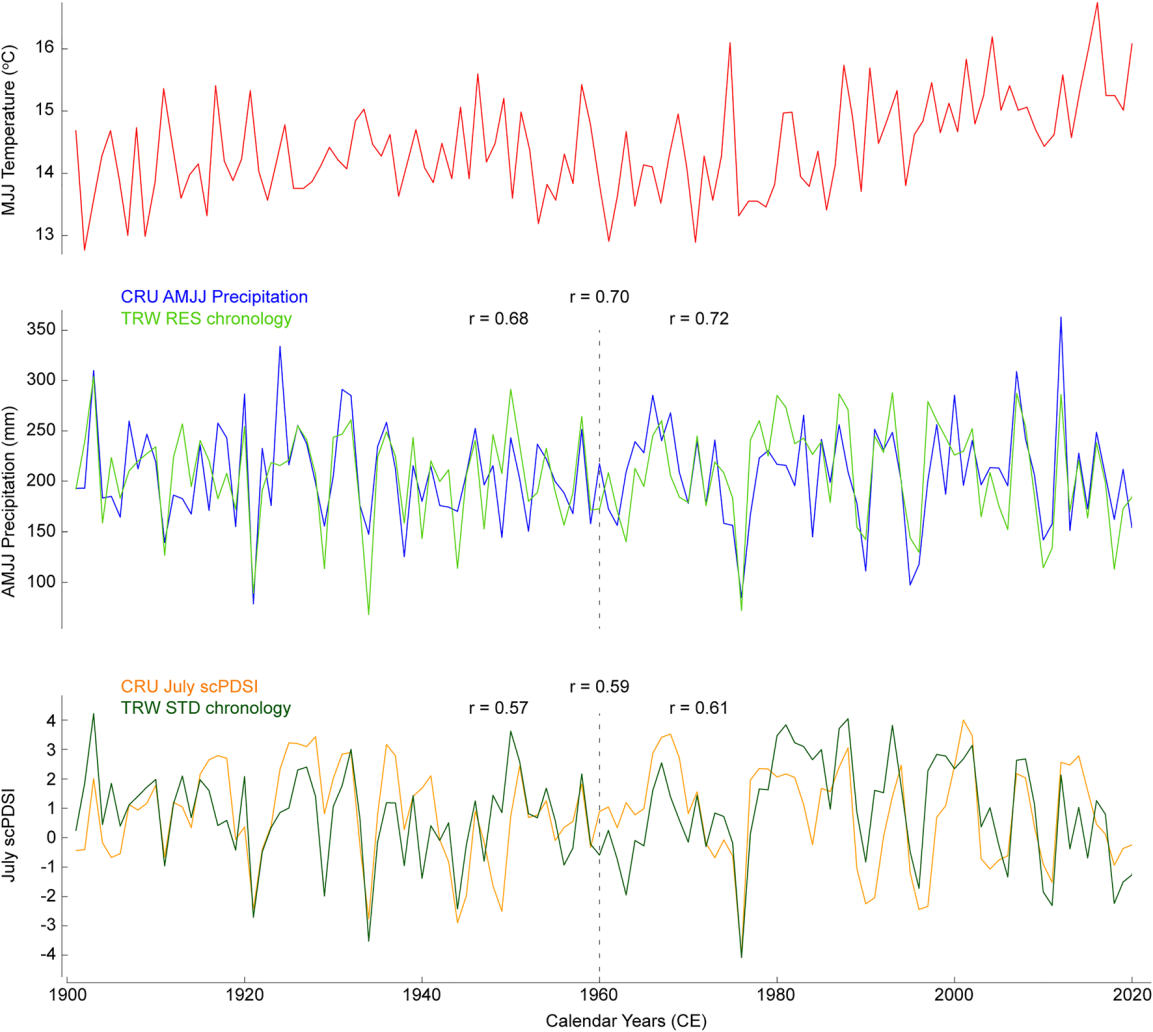
Proxy-target agreement. Gridded (51−52°N, 2°W−1°E) instrumental MJJ temperature (red), AMJJ precipitation (blue), and July scPDSI series (orange) with scaled residual (light green) and standard (dark green) yew TRW chronologies. The correlations between the target and the proxy data are given for the full period 1901−2020 and two early/late split periods, 1901−1960 and 1961−2020

Spatial correlation maps reveal the agreement between the proxies and climate targets extends over northwest Europe (Fig. 5). For the full 1901−2020 period of instrumental gridded data, the strongest correlations > 0.6 are found across southern England and northwest France, decreasing to 0.4 over central France, the Low Countries, and central Germany, and fading to 0.2 in southern France, the Alps, Czech Republic, and central Poland. In the late split period 1961−2020, both parameters reveal higher correlations > 0.4 across entire Germany and northern Europe. An intensified agreement in the late split period is also observed when correlating the proxies against the targets averaged over the 51−52°N, 2°W−1°E grid (Fig. 6). The RES chronology correlates at 0.70 with AMJJ precipitation in the full period but at 0.68 and 0.72 in the early and late split periods, respectively. The correlation between the STD chronology and July scPDSI is 0.59 in the full period and 0.57 and 0.61 in the early and late split periods, respectively. Both proxies scaled to the targets have a stable variance and successfully capture the range of target variability (Fig. 6).

**Fig. 7:**
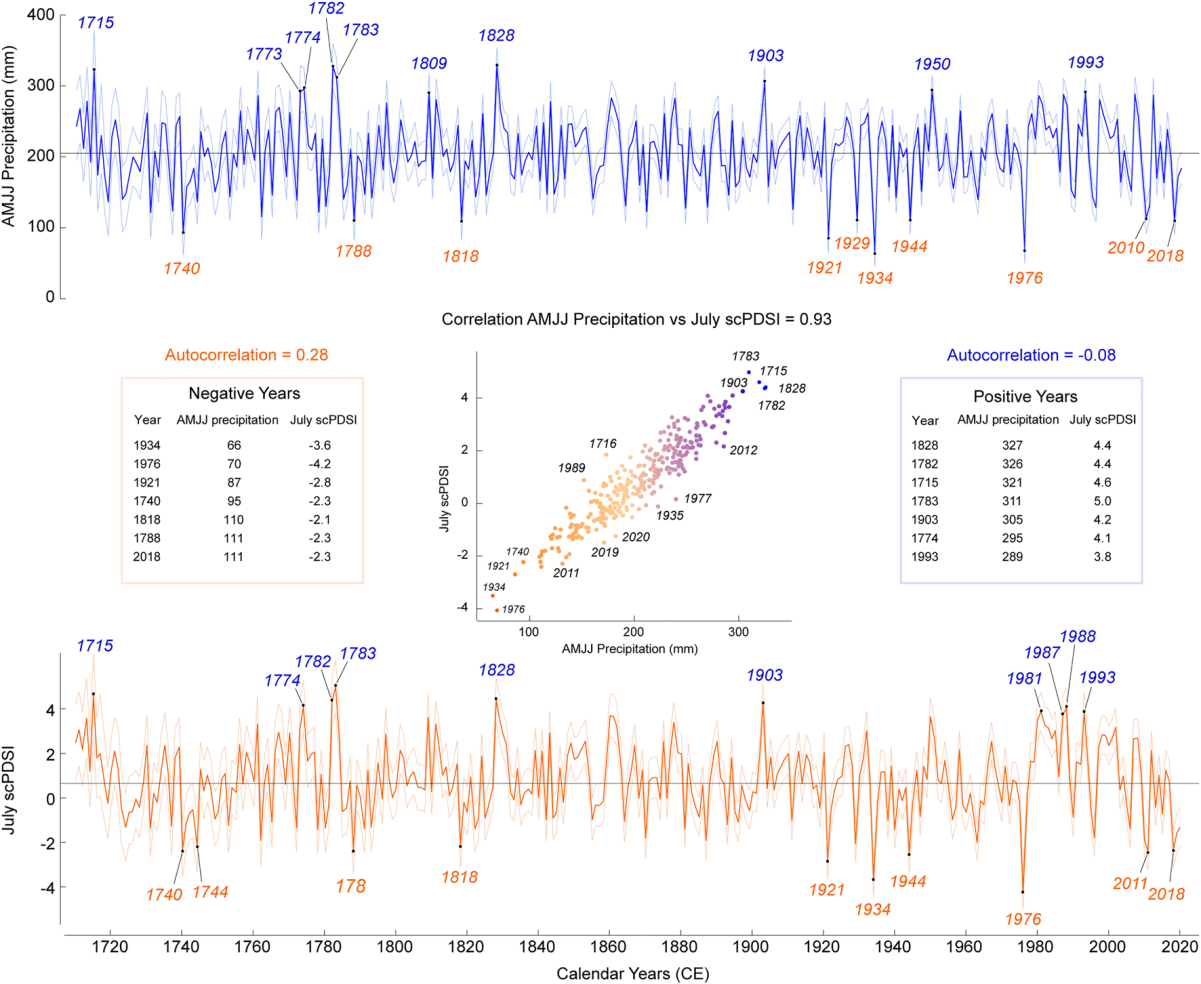
Reconstructions of southern England AMJJ precipitation totals (mm, upper panel) and July scPDSI (lower panel) for the period 1710−2020 CE. Uncertainties are computed as a composite of three error sources: sample replication, chronology building, and calibration. The ten wettest and ten driest years are marked at each reconstruction, and the most extreme years according to both reconstructions are listed. The scatter plot shows the relation between the reconstructed AMJJ precipitation and July scPDSI with the most extreme years labelled

The two pseudo-independent hydroclimate reconstructions of AMJJ precipitation and July scPDSI for southern England span 1710−2020 CE (Fig. 7), correlate with each other at 0.93, and have first-order autocorrelations of −0.08 and 0.28, respectively. The AMJJ precipitation totals range between 65 and 330 mm, with a mean of 205 mm, while July scPDSI varies between −4.2 and 5.0, with a mean of 0.8. Each reconstruction has 11 negative extremes with values below 1.8 standard deviations, out of which nine are shared: 1740, 1788, 1818, 1921, 1929, 1934, 1944, 1976, and 2018. The years 1762 and 2010 appear extremal in the AMJJ precipitation reconstruction only, and the years 1744 and 2011 are below the threshold in the July scPDSI reconstruction only. Extreme droughts cluster between the 1920s and the 1970s, but considering a 30-year window and a threshold of 1 standard deviation, strong droughts were occurring most frequently between the 1760s and the 1790s. There are six positive years with values above 1.8 standard deviation in both chronologies: 1715, 1774, 1782, 1783, 1828, and 1903. In addition, the years 1773 and 1950 are above the threshold in the AMJJ precipitation reconstruction, and the years 1860, 1981, 1987, 1988, and 1993 are pluvial extremes in the July scPDSI reconstruction. Overall, drier conditions were persistent from the 1720s till the 1750s and from the 1910s till the 1950s, and wetter conditions dominated from the 1970s till the 2010s.

## Discussion

Yew trees are known for slow growth (Thomas and Polwart 2003), a feature that enables their longevity (Issartel and Coiffard 2011). Although the AGR in our dataset (1.4 mm) is lower than that of many other conifer species (Cedro 2023), it is almost twice that of yews growing in eastern England during the mid-Holocene (Bebchuk et al. 2024). Especially wide rings contributing to high AGR have been formed since the 1970s (Fig. 2) and are not associated with an age-related trend (Fig. S1). Although the high-frequency variability of yew TRW is primarily explained by precipitation, the long-term increase in yew growth rate is likely associated with rising spring–summer temperatures (Fig. 6). We therefore note that current anthropogenic warming has accelerated yew growth and might reduce the trees’ lifespan, since increased biomass productivity decreases tree longevity: grow fast—die young (Büntgen et al. 2019).

The observed high dependence of yew trees on precipitation and soil moisture availability in the temperate maritime climate of England is unexpected. Although some yews were sampled in gardens and churchyards (Table 2), where irrigation might take place, their precipitation sensitivity remains strong. Our results suggest that yew trees experience moisture deficit, which is driven by either little precipitation or high evaporation rates and intensified due to the species’ shallow root system (Bebchuk et al. 2024; Thomas and Polwart 2003). Therefore, we conclude that the climate of southern England is not optimally humid for yew trees, and irrigation, if such, is delayed or insufficient to compensate for dry spring and summer conditions.

High correlations of yew TRWs with precipitation totals and drought indices introduce *Taxus baccata* as a new proxy for hydroclimate reconstructions. Moreover, the strong precipitation signal found in yews from southern England opens the possibility of developing precipitation reconstructions in regions with humid climates, such as the British Isles, which are traditionally overlooked in tree-ring research (Büntgen et al. 2010). Yew longevity and a possibility to cross-date even decayed yew wood provide the potential for building long yew chronologies and thus extending climate reconstructions further back in time (Hindson and Moir 2023; Moir et al. 2013). The recent discovery of hundreds of sub-fossil yew trunks in eastern England suggests the possibility to reach back into the mid-Holocene (Bebchuk et al. 2024).

Our AMJJ precipitation and July scPDSI reconstructions are based on the same TRW dataset and are, therefore, referred to as ‘pseudo-independent’. We used the RES TRW chronology, which contains high-frequency variability, for reconstructing precipitation totals, and the STD TRW chronology, which preserves longer-term variability, for reconstructing scPDSI. Since the scPDSI incorporates precipitation amounts (van der Schrier et al. 2013), the target parameters are not independent, thus the reconstructions are expected to share a common signal. Our targets correlate with each other at 0.58 and the reconstructions correlate at 0.93, suggesting precipitation plays a more important role than temperature in defining scPDSI in the humid oceanic climate of southern England. Meanwhile, the difference between our pseudo-independent reconstructions is meaningful, since the residuals of the reconstructions correlate significantly (*p* < 0.05) with July scPDSI target at 0.3. The difference in the reconstructions is explained by the temperature share in scPDSI. This study demonstrates that tree-ring chronologies can hold more than one climate signal, and it is a researcher’s choice of treatments applied to the data that determines which climate signal at which frequencies will be extracted.

Our new reconstructions reflect both positive and negative hydroclimate extremes. Of the 20 driest (wettest) years in the CRU 1901−2020 record, our AMJJ precipitation reconstruction identifies 11 (9) extremes, and the July scPDSI reconstruction identifies 9 (5) (Fig. 6). A few years in the instrumental 1901−2020 period were clearly over- and underestimated by the yew reconstructions. For instance, precipitation and scPDSI were overestimated by 20% and 50%, respectively, in 1950 marking it one of the wettest years in the calibration period. Conversely, the year 1934 was reconstructed as one of the driest in the calibration period, yet precipitation and scPDSI were underestimated by 56% and 38%, respectively. In contrast, some extreme events were not fully captured, such as the pluvial years 1924 and 2012, and the dry years 1949 and 1990. The discrepancy between proxy reconstructions and target data is likely an effect of unavoidable “noise” that includes biotic and abiotic factors influencing yew growth. Nevertheless, our yew reconstructions capture the majority of dry and pluvial spells, even when compared with long instrumental records available for the UK (Fig. S2), including southeast England precipitation (from 1873), south England precipitation (from 1836), and England & Wales precipitation (from 1766) (Alexander and Jones 2001; Gregory et al. 1991; Jones and Conway 1997; Wigley and Jones 1987). A few extremes not captured by our yew reconstructions are those found in the beginning of the longest instrumental records: droughts in 1844, 1868, and 1870 in the south England precipitation series, and pluvials in 1768, 1777, 1779, 1789, and 1792 in the England & Wales precipitation series. These discrepancies are likely attributed to artefacts in early meteorological observations, the distance that separates our trees from meteorological stations, and in the case of the latter record, a probable maritime effect.

Our two pseudo-independent reconstructions correlate with other hydroclimate reconstructions from England between 0.3 and 0.5 (Table S1). *Taxus* captures the magnitude of precipitation variability more accurately and has a larger spatial extent of high correlations with the target (Fig. S3) than that of the regional oak TRW precipitation reconstructions (Cooper et al. 2013; Wilson et al. 2013). This difference is likely due to both yew's high sensitivity to precipitation and oak's inability to track dry and pluvial extremes (Cooper et al. 2013; Wilson et al. 2013). Conversely, the OWDA has a larger extent of strong spatial correlations than our scPDSI reconstruction (Fig. S4). This is an expected result since the OWDA is a gridded reconstruction combining the oak TRW precipitation reconstructions for England together with other hydroclimate reconstructions.

According to our yew reconstructions, the lowest frequency of droughts over the past 310 years occurred between the 1790s and the 1820s, a period characterised by increased storminess in the English Channel (Cornes and Jones 2024), while the highest drought frequency is found in the second half of the eighteenth century (Fig. 7). These results suggest that the frequency of droughts in the past three decades of anthropogenic warming is still within the range of natural hydroclimate variability. The anomalous dry conditions at the end of the eighteenth century, surpassing recent extremes, are also found in hydroclimate reconstructions for western France by Masson-Delmotte et al. (2005), soil moisture reconstruction for western Europe by Wang's et al. (2022), and precipitation reconstructions for England by Cooper et al. and Wilson et al. (2013). Meanwhile, TRSI-based hydroclimate reconstructions for central and western Europe suggest the intensity of recent summer droughts is unprecedented (Büntgen et al. 2021; Freund et al. 2023). We therefore argue that understanding recent drought extremes in the context of long-term hydroclimate variability requires a multi-species and multi-proxy approach.

Although the frequency of recent droughts does not appear unprecedented in our yew reconstructions, the scPDSI shows a decreasing trend since the 1990s (Fig. 7). Moreover, the sensitivity of yew trees to precipitation has increased in the past decades (Fig. 5). We suggest this drying trend is triggered by rising temperatures, increased evaporation, and wider synoptic weather patterns such as the North Atlantic Oscillation (NAO) and East Atlantic pattern (EA). In southern England, a combination of a positive NAO phase and a negative EA phase is linked to decreased precipitation in both summer and winter (West et al. 2019), as the storm track in the North Atlantic moves north, and easterly winds transport warm continental air from mainland Europe (Linderholm et al. 2009). The positive NAO phase observed since 1986, and forecasted to stay positive (West et al. 2022), may further intensify the drying trend, exacerbating plant drought stress and showing a cascade of consequences that can result from large-scale alterations in climate dynamics.

## Supplementary Information

Below is the link to the electronic supplementary material.

Supplementary file1

## Data Availability

Raw tree-ring width measurements will be available at the International Tree Ring Data Bank.
