# Supplementary material for: Taxus tree-ring chronologies from southern England reveal western European hydroclimate changes over the past three centuries
Source: Clim Dyn. 2025 Jan 30;63(2):108. doi: 10.1007/s00382-025-07601-2 (PMC11782366; doi:10.1007/s00382-025-07601-2)
Supplement: Supplementary file 1 — Supplementary file1 [file 382_2025_7601_MOESM1_ESM.docx]

# **Supplementary**

|  | MAMJJ precipitation,  Cooper et al., 2013 | MAMJJ precipitation,  Wilson et al., 2013 | AMJJ precipitation, this study | JJA scPDSI, OWDA  Cook et al., 2015 |
| --- | --- | --- | --- | --- |
| MAMJJ precipitation | 0.60 |  |  |  |
| AMJJ precipitation | 0.30 | 0.42 |  |  |
| JJA scPDSI | 0.61 | 0.65 | 0.51 |  |
| July scPDSI, this study | 0.30 | 0.44 | 0.93 | 0.49 |

Table S1. Correlation matrix of oak-based MAMJJ precipitation reconstructions by Cooper et al. and Wilson et al. (2013), AMJJ precipitation reconstruction developed in this study, JJA scPDSI reconstruction from Old World Drought Atlas (OWDA) by Cook et al. 2015, and July scPDSI reconstruction developed in this study.


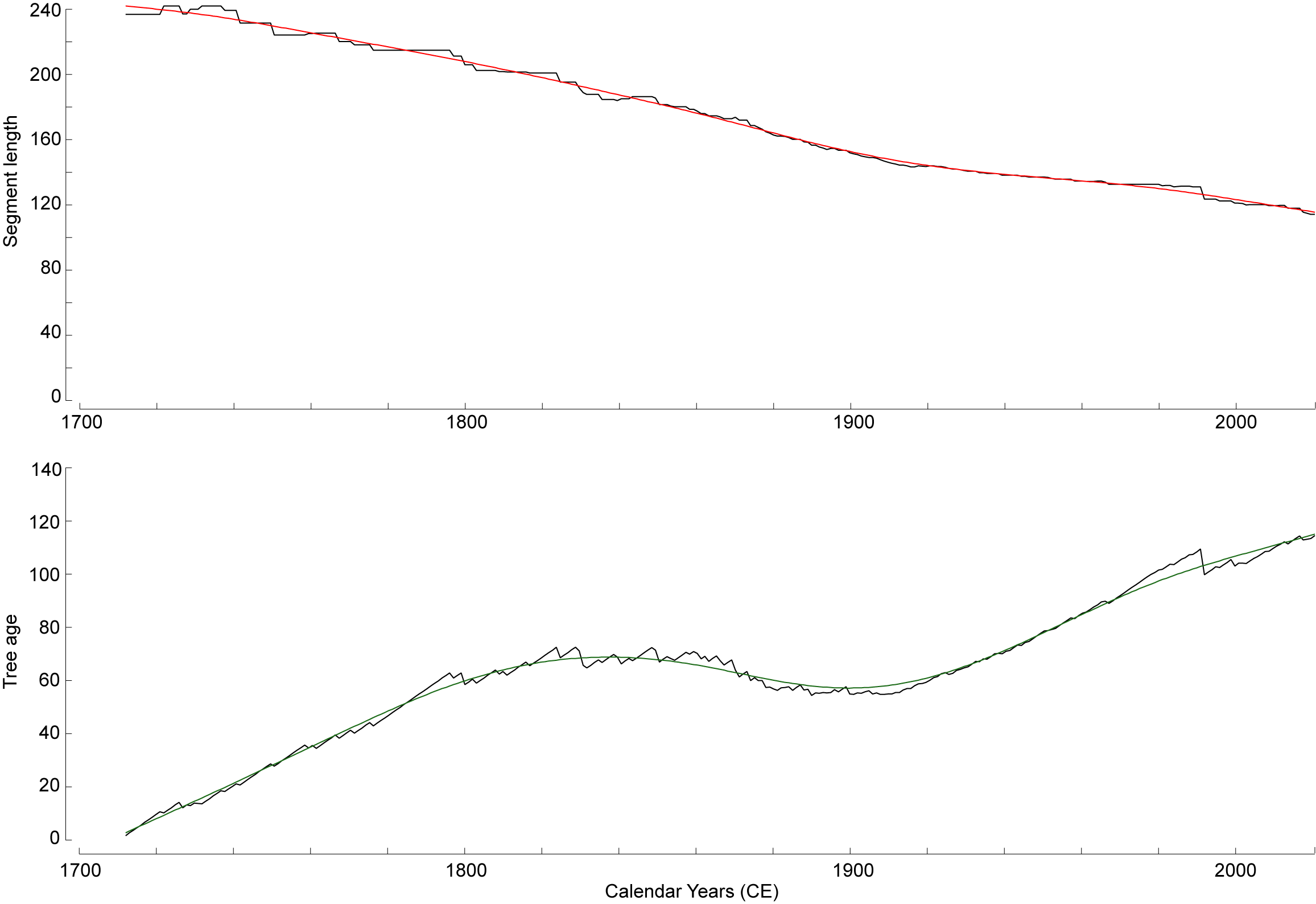


Figure S1. Mean segment length (in the upper panel) and mean tree age (in the lower panel) of the yew dataset.


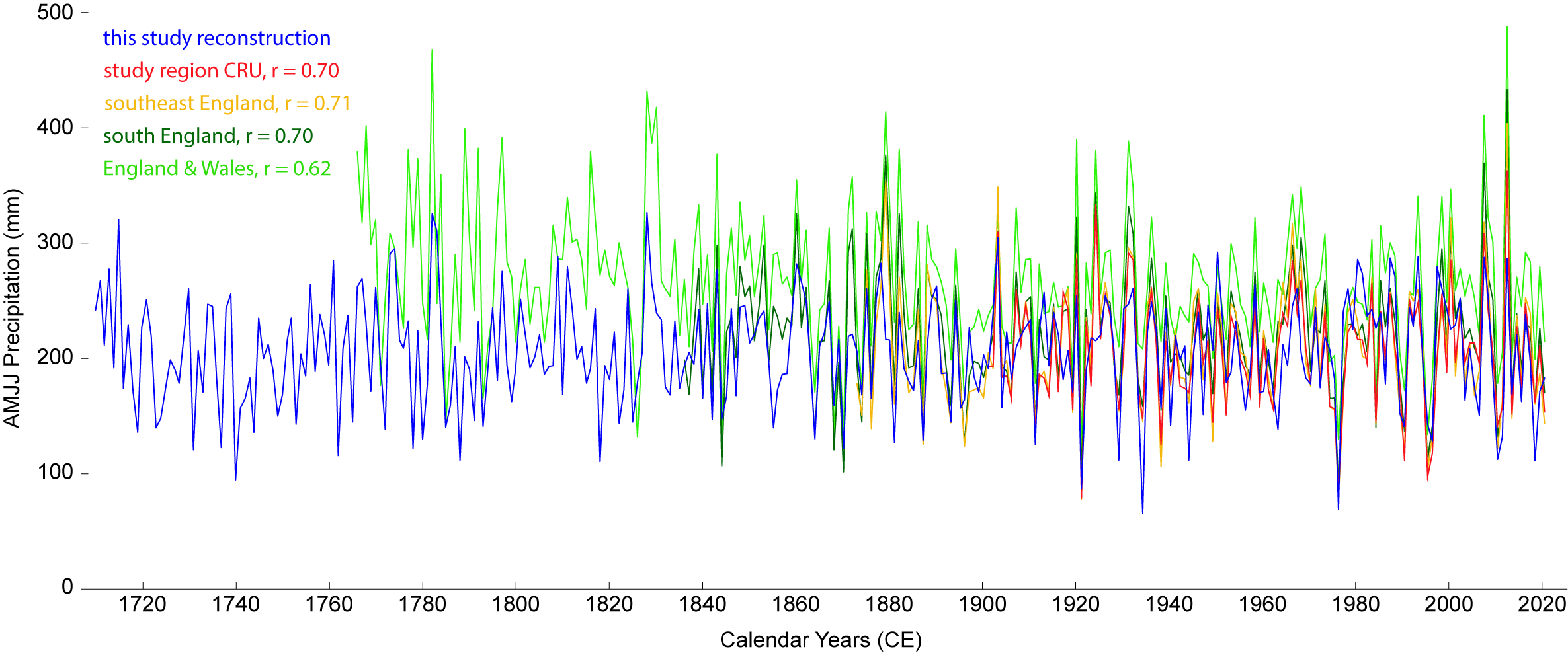


Figure S2. Yew AMJJ precipitation reconstruction (in blue) in the context of instrumental precipitation records for England: CRU series over the study region starting in 1901 (in red), southeast England precipitation series starting in 1873 (in orange), south England precipitation series starting in 1836 (in dark green), and England & Wales precipitation starting in 1766 (in light green) (Alexander and Jones, 2001; Gregory et al., 1991; Jones and Conway, 1997; Wigley and Jones, 1987). Pearson’s correlation coefficients are computed between the reconstruction and each measurement series.


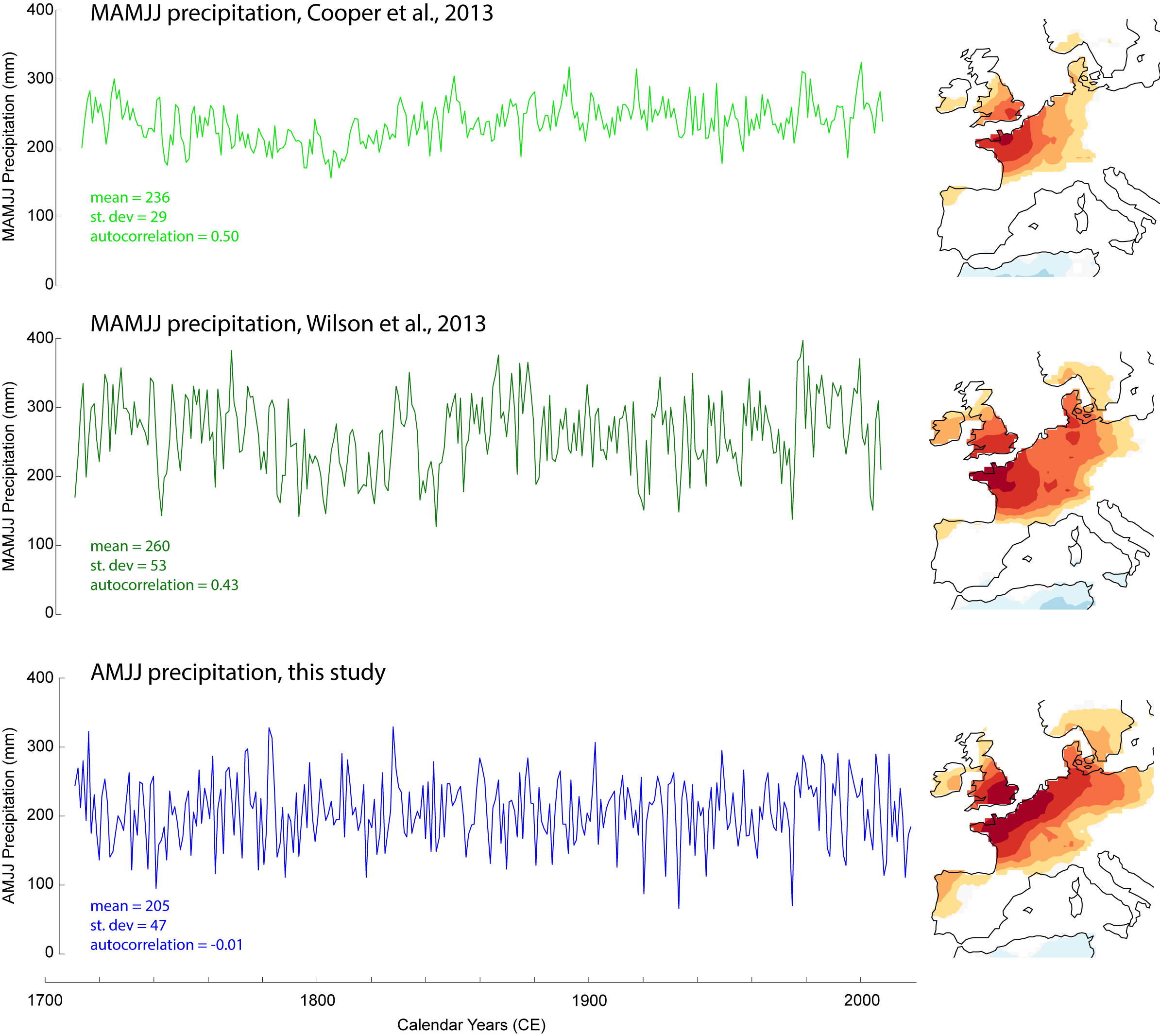


Figure S3. Oak TRW precipitation reconstructions by Cooper et al. (2013) in the upper panel and Wilson et al. (2013) in the middle panel, and the yew precipitation reconstruction developed in this study in the lower panel. Spatial correlation maps are computed against CRU TS 4.07 0.5×0.5 data, for the colour legend refer to the Figure 5.


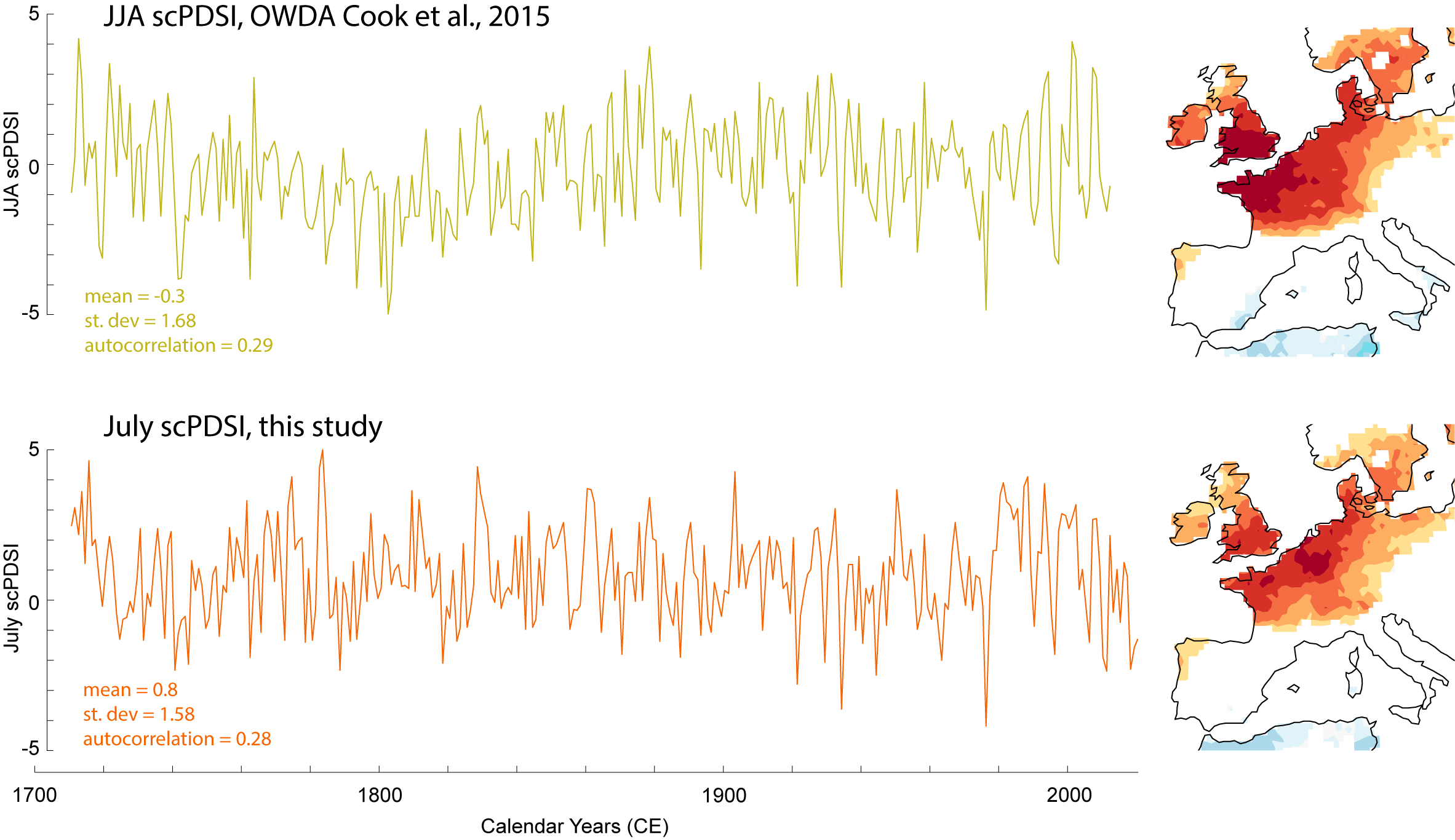


Figure S4. Self-calibrating Palmer Severity Drought Index (scPDSI) reconstruction from the Old World Drought Atlas (OWDA) by Cook et al. (2015) in the upper panel and the scPDSI reconstruction developed in this study in the lower panel. Spatial correlation maps are computed against CRU TS 4.07 0.5×0.5 data, for the colour legend refer to the Figure 5.
